# Maintaining endotracheal tube cuff pressure at 20 mm Hg to prevent dysphagia after anterior cervical spine surgery; protocol of a double-blind randomised controlled trial

**DOI:** 10.1186/1471-2474-14-280

**Published:** 2013-09-25

**Authors:** Mark P Arts, Thijs C D Rettig, Jessica de Vries, Jasper F C Wolfs, Bas A in’t Veld

**Affiliations:** 1 Department of Neurosurgery, Medical Center Haaglanden, PO Box 432, 2501 CK The Hague, The Netherlands; 2 Department of Anesthesiology, Medical Center Haaglanden, The Hague, The Netherlands

**Keywords:** Anterior cervical spine surgery, Surgical retractor, Endotracheal tube cuff pressure, Sore throat, Hoarseness, Dysphagia

## Abstract

**Background:**

In anterior cervical spine surgery a retractor is obligatory to approach the spine. Previous studies showed an increase of endotracheal tube cuff pressure after placement of a retractor. It is known that high endotracheal tube cuff pressure increases the incidence of postoperative dysphagia, hoarseness, and sore throat. However, until now no evidence supports the fact whether adjusting the endotracheal tube cuff pressure during anterior cervical spine surgery will prevent this comorbidity. We present the design of a randomized controlled trial to determine whether adjusting endotracheal tube cuff pressure after placement of a retractor during anterior cervical spine surgery will prevent postoperative dysphagia.

**Methods/design:**

177 patients (aged 18–90 years) scheduled for anterior cervical spine surgery on 1 or more levels will be included. After intubation, endotracheal tube cuff pressure is manually inflated to 20 mm Hg in all patients. Patients will be randomized into two groups. In the control group endotracheal tube cuff pressure is not adjusted after retractor placement. In the intervention group endotracheal tube cuff pressure after retractor placement is maintained at 20 mm Hg and air is withdrawn when cuff pressure exceeds 20 mm Hg. Endotracheal tube cuff pressure is measured after intubation, before and after placement and removal of the retractor. Again air is inflated if cuff pressure sets below 20 mmHg after removal of the retractor. The primary outcome measure is postoperative dysphagia. Other outcome measures are postoperative hoarseness, postoperative sore throat, degree of dysphagia, length of hospital stay, and pneumonia. The study is a single centre double blind randomized trial in which patients and research nurses will be kept blinded for the allocated treatment during the follow-up period of 2 months.

**Discussion:**

Postoperative dysphagia occurs frequently after anterior cervical spine surgery. This may be related to high endotracheal tube cuff pressure. Whether adaptation and maintaining the pressure after placement of the retractor will decrease the incidence of dysphagia, has to be determined by this trial.

**Trial registration:**

Netherlands Trial Register (NTR) 3542: http://www.trialregister.nl.

## Background

The surgical procedure in patients undergoing anterior cervical spine surgery can only be performed by placement of a retractor system in order to expose the anterior cervical spine. This retractor is placed between the carotic sheath laterally, and the trachea and esophagus medially. Previous studies showed that after positioning and opening the retractor, the endotracheal tube (ETT) cuff pressure increases significantly [1,2]. Structures trapped between the retractor and the endotracheal tube cuff, like the recurrent laryngeal nerve, may be compromised. In patients who were treated with anterior cervical discectomy and fusion, the incidence of recurrent laryngeal nerve injury is about 2%, irrespective of the side of approach [3]. Consequently, dysphagia, hoarseness, and sore throat may occur. These complications occur in other types of surgery as well and may be the result of endotracheal intubation or placement of a laryngeal mask. The overall incidence of postoperative dysphagia after general anesthesia varies from 16 to 60%, hoarseness 21 to 51%, and sore throat from 21 to 74% [1,4-8]. Suggested mechanisms are neuropraxia of the recurrent laryngeal nerve, mucosal injury, postoperative hematoma or edema.

Previous studies have shown that maintaining ETT cuff pressure between 15 and 25 mm Hg can reduce endotracheal intubation-related complications in patients undergoing general anesthesia [9,10]. With regards to anterior cervical spine surgery, lowering the ETT cuff pressure after placement of the retractor may influence postoperative dysphagia, sore throat, and hoarseness. However, a randomized controlled trial showed that pressure adjustment did not reduce the incidence of vocal cord immobility [11]. A small study from 2002 analysed patients undergoing anterior cervical spine surgery and data suggested a lower incidence of sore throat in patients with lower cuff pressures, while a recently performed observational study showed less recurrent laryngeal nerve palsy in patients with reduction of ETT cuff pressure [5,12]. However, until now, adjusting ETT cuff pressure after placement of surgical retractor is not generally performed.

We will evaluate whether ETT cuff pressure adjustment after retractor placement has any influence on postoperative dysphagia, sore throat, and hoarseness. The results of this study may lead to standard adjustment of ETT cuff pressure in all patients undergoing anterior cervical spine surgery.

## Methods/design

We designed a single center, observer and patient blinded randomized controlled trial to investigate whether adjustment of ETT cuff pressure in anterior cervical spine surgery will lead to reduction of postoperative dysphagia, hoarseness and sore throat. The follow-up period is 2 months. The protocol is approved by the medical ethics committee Zuidwest Holland, The Netherlands (number NL35829.098.11). The study will be performed in accordance with the guidelines for good clinical practice.

### Patients

All patients between 18 and 90 years of age requiring primary anterior cervical spine surgery are eligible for this trial. Additional inclusion and exclusion criteria are listed in Table 1. During the patient’s visit to the preoperative outpatient clinic, the anesthesiologist decides whether the patient is eligible for this trial, conform the selection criteria. The study will be explained and, in case of a positive reaction, patients will receive written information. A research nurse will contact the patient a few days after the visit to the outpatient clinic. Informed consent will be obtained just prior to the operation.

**Table 1:** Selection criteria for trial eligibility

	
**Inclusion criteria**	
	● Age 18-90 years
	● Primary anterior cervical spine surgery on 1 or more levels
	● Informed consent
**Exclusion criteria**	
	● Pre-operative dysphagia, hoarseness or sore throat
	● Pre-operative recurrent laryngeal nerve palsy
	● Previous anterior cervical spine surgery
	● Inadequate Dutch language
	● Planned fiberoptic intubation or rapid sequence induction
	● Peroperative use of N_2_O
	● Mentally disabled patients

### Randomization procedure

Subjects will be screened for inclusion into the study and eligible subjects will be randomized to either the intervention or the control group, according to a pre-determined randomization schedule. This randomization was done, using an online randomization program (http://www.randomization.com), using the method of 4 randomly permuted blocks.

In the operating room, after induction of general anesthesia, the anesthesiologist will open an envelope and the allocated treatment will be performed. Patients and research nurses will be kept blinded for the allocated treatment during the follow-up of 2 months.

### Intervention

Patients will be randomized into adjustment of the ETT cuff pressure after retractor placement (intervention group), or into no adjustment of the ETT cuff pressure after retractor placement (control group). The type of general anesthesia is determined by the attending anesthesiologist. Intubation is performed by a skilled laryngoscopist with more than 1 year experience. To minimize baseline dysphagia after conventional intubation with a Macintosh laryngoscope, the Glidescope® is used. Endotracheal tube size is 7.0 mm for women and 8.0 mm for men. After intubation, ETT cuff pressure is manually inflated to 20 mm Hg in all study patients. In the control group, no ETT cuff pressure intervention is performed after placement of the retractor. In the intervention group, the ETT cuff pressure is maintained at 20 mm Hg after placement and after removal of the retractor. Air is withdrawn when cuff pressure exceeds 20 mm Hg. In the absence of a device to measure ETT cuff pressure continuously, cuff pressure is measured at several time points: after intubation, before and after placement and removal of the retractor from the start of induction to the end of anesthesia by use of a manometer. The mean intraoperative pressure will be calculated from the values measured directly after insertion of the retractor and just before removal of the retractor. In addition, next to standard patient data, time of retraction, duration of operation, level of operation, number of levels operated on, the use of a cage or other implant, the attending neurosurgeon, and the dose of neuromuscular blocking agent are recorded.

### Surgical procedure

All patients will be positioned supine with their neck in neutral position or slightly extended under general anaesthesia. The affected cervical disc level will be verified with fluoroscopy. A small transverse incision will be made on the right side in all patients. The prevertebral space is approached between the carotic sheath laterally, and the trachea and oesophagus medially. The Caspar Cervical Retractor System (CCR) (Braun Aesculap) or the Trimline retractor (Medtronic) are used in all patients. Depending on the pathology of the patients, the following surgical procedures are performed: 1 level anterior cervical disectomy with interbody fusion (ACDF) or disc prosthesis, 2 level ACDF, or corpectomy with placement of expandable cage.

### Baseline data

Baseline assessment includes age, gender, body mass index, medical history and comorbidity, number of levels operated on, duration of operation, duration retractor placement, and use of cervical implants.

### Outcome measures

The primary outcome measure is postoperative dysphagia documented with the Bazaz dysphagia scale (Table 2) [13]. Secondary outcome measures are postoperative hoarseness, sore throat, degree of dysphagia, incidence of pneumonia, and length of hospital stay. Hoarseness will be scored using a clinician-based voice assessment protocol; Grade, Roughness, Breathiness, Asthenia, Strain (GRBAS) and by using a numeric rating scale (NRS) [14]. A sore throat will be recorded using a numeric rating scale (NRS 0–10).

**Table 2:** Bazaz dysphagia scale

**Do you experience difficulty swallowing?**	
None	No episode of swallowing difficulty.
Mild	Experienced only rare episodes of dysphagia and not considered a significant problem.
Moderate	Occasional swallowing difficulty with specific foods (e.g., bread, steak).
Severe	Frequent difficulty swallowing (e.g., with the majority of foods).

### Outcome assessment

We will assess the above described outcome parameters on two separate occasions; 24 hours following the operation, and 2 months after surgery. Patients are questioned about the presence of dysphagia, hoarseness and sore throat by a blinded observer. Both interviews will be recorded on tape using a high quality digital recorder.

### Sample size

The sample size calculation is based on the hypothesis that the incidence of dysphagia in the intervention group is 20% less than in the control group (40%). Assuming a power of 80%, 82 patients have to participate in each arm. Including 8% loss to follow-up, a total of 177 patients need to be included.

### Data analysis

All patients will be randomized in a 1:1 ratio to receive either standard care or adapted cuff pressure. To investigate the association between the presence and severity of dysphagia and cuff pressure, we will compare the two groups by means of chi-square statistics or if necessary with ANOVA. In addition we will use multivariate logistic regression analysis to adjust for potential confounding. A p-value of less than 0.05 will be considered significant. SPSS (version 16.0 or higher SPSS Inc. Chicago, IL) will be used for calculations. Univariate analysis will be performed to identify factors related to dysphagia and secondary outcomes.

Although the numbers of participants are relatively low, we will perform multivariate analysis to evaluate factors that might modify the effect of cuff pressure on various outcome parameters. These factors are determined before analysis and include gender, smoking, duration of surgery, level of surgery, extent of surgery (1 level versus 2 or more levels), and type of implant.

## Discussion

The presented study is a randomized controlled double blind trial in which we will evaluate whether ETT cuff pressure adjustment after retractor placement has any influence on postoperative dysphagia, hoarseness and sore throat in patients undergoing anterior cervical spine surgery. Possibly, the development of this postoperative morbidity may not only be caused by surgical manipulation, but neuropraxia of the recurrent laryngeal nerve may also be related to high ETT cuff pressure. Therefore, the results of this study may lead to standard adjustment of ETT cuff pressure in every patients undergoing anterior cervical spine surgery in order to decrease postoperative morbidity. Recruitment of patients has started in December 2011 and will be finished when 177 patients have been included, which we expect to be at the end of 2013.

## Abbreviations

ETT: Endotracheal tube; GRBAS: Grade, Roughness, Breathiness, Asthenia, Strain; NRS: Numeric rating scale.

## Competing interests

The authors declare that they have no competing interests.

## Authors’ contributions

TR, JV, MA and BV designed the protocol. MA and JW performed all surgical procedures. BV is responsible for sample size calculation and randomization procedure. BV is the principal investigator. All authors participated in the trial design and coordination. All authors read and approved the final manuscript.

## Pre-publication history

The pre-publication history for this paper can be accessed here:

http://www.biomedcentral.com/1471-2474/14/280/prepub
